# Transcutaneous auricular nerve stimulation modulates the functional connectivity of the descending pain modulation system and reward network in patients with chronic low back pain

**DOI:** 10.1016/j.neurot.2025.e00611

**Published:** 2025-06-02

**Authors:** Tingting Li, Yuefeng Wu, Yuanyuan Li, Sierra Anne Hodges, Sveta Reddy, Lucy Chen, Valeria Sacca, Jian Kong

**Affiliations:** Department of Psychiatry, Massachusetts General Hospital, Harvard Medical School, Charlestown, MA 02129, USA

**Keywords:** Chronic low back pain, Transcutaneous auricular vagus nerve stimulation (taVNS), Transcutaneous greater auricular nerve stimulation (tGANS), Functional connectivity, Descending pain modulation system

## Abstract

This study aims to examine the modulatory effects of transcutaneous auricular vagus nerve stimulation (taVNS) on Chronic low back pain (cLBP). 70 cLBP patients were recruited and randomized into taVNS or transcutaneous greater auricular nerve stimulation (tGANS) group. Both interventions were administered by participants themselves after initial training (five times/week for four weeks). Magnetic resonance imaging (MRI) data were collected at baseline and after 4-week interventions. Seed-based static and dynamic functional connectivity (sFC and dFC) were performed to investigate the modulation effects on descending pain modulation system and reward network using the periaqueductal gray (PAG) and ventral tegmental area (VTA) as seeds. 51 patients (taVNS: n ​= ​25; tGANS: n ​= ​26) completed the study. Within-group comparisons showed a significant improvement in pain-related outcomes for both groups. Between-group comparisons revealed no significant differences. FC analysis showed that both taVNS and tGANS can increase the PAG - postcentral gyrus sFC. The taVNS is associated with increased PAG - amygdala and PAG - paracentral gyrus and decreased PAG – medial frontal cortex sFCs compared to tGANS. The present study suggest that both taVNS and tGANS can alleviate cLBP through distinct yet overlapping pathways. Our findings underscore the potential of auricular nerve stimulation as a telehealth solution for cLBP and other chronic pain conditions.

## Introduction

Chronic low back pain (cLBP) is a prevalent clinical condition and the leading cause of long-term disability among middle aged individuals worldwide [1,2]. Despite frequent referrals for comprehensive multidisciplinary treatments [3], these interventions often fail to achieve consistent and significant reductions in pain intensity [4,5], highlighting the need for more treatment options.

Non-invasive transcutaneous auricular vagus nerve stimulation (taVNS) has emerged as a promising treatment for various pain disorders. Unlike traditional vagus nerve stimulation (VNS), which involves surgical implantation, taVNS stimulates the cutaneous distribution of the auricular branch of the vagus nerve (ABVN) on the external ear. The non-invasive nature has inspired extensive research into its diverse applications [6], including cLBP [7], migraine [8,9], depression [[10], [11], [12]], anxiety [13], insomnia [14] and many other conditions. Furthermore, given its feasibility for self-administration following appropriate training, taVNS also holds the potential for broader telehealth implementation.

The mechanisms underlying taVNS are still being explored. One hypothesis suggests that taVNS modulates the activity and/or connectivity of multiple brain regions including key regions of the descending pain modulation system (DPMS) - such as the periaqueduct gray (PAG), prefrontal cortex, amygdala, and anterior cingulate cortex (ACC) [15]– as well as the reward network, including the ventral striatum [16]. These findings establish a foundation for the potential of taVNS in pain management.

Recently, the interaction of the DPMS and reward network in pain management has gained attention. Studies have shown that the DPMS and the reward network are interconnected pathways that jointly influence pain perception and behavior. The DPMS, with the PAG as the hub, receiving signals from the ACC, prefrontal cortex, and amygdala, modulates pain signals at the spinal cord level, either inhibiting or amplifying them. The reward network, involving regions like the ventral tegmental area (VTA) and nucleus accumbens, mediates pleasure and motivation. Positive or rewarding experiences trigger dopamine release within the reward network, which can activate descending pain inhibition and reduce pain perception. In chronic pain, diminished reward sensitivity contributes to worsened pain perception and impaired coping mechanisms, highlighting the need to better understand and target these systems in therapeutic strategies [[17], [18], [19]].

In this study, we investigated the modulatory effects of 4-week taVNS as compared to transcutaneous greater auricular nerve stimulation (tGANS) at the earlobe on the DPMS and reward network in cLBP patients, using both static and dynamic resting state functional connectivity (FC) methods. We hypothesize that taVNS would modulate the FC of the PAG and the VTA, thereby significantly alleviating back pain and regulating the reward and pain modulation systems.

## Materials and methods

### Participants

Seventy-one participants diagnosed with non-specific cLBP [20] for at least six months [21] were enrolled and randomly assigned in a 1:1 ratio to receive either taVNS or tGANS treatment. The study's protocol received approval from the Institutional Review Board (IRB) at Massachusetts General Hospital, and all experimental procedures adhered to the IRB's ethical guidelines for the protection of human subjects. All participants provided informed consent before commencing the experiment. Detailed inclusion and exclusion criteria are presented in the supplementary materials. The study was registered on ClinicalTrials.gov (NCT03959111).

### Experimental procedures and clinical assessment

This study was a single-blinded, randomized controlled clinical trial. Randomization codes were generated using *R* software (version 3.3.4), ensuring each participant had an equal probability of being assigned to either the taVNS or tGANS group. Each participant attended two MRI scans: one before and one after the four-week intervention. After randomization, participants received training on how to administer either taVNS or tGANS.

**Fig. 1:**
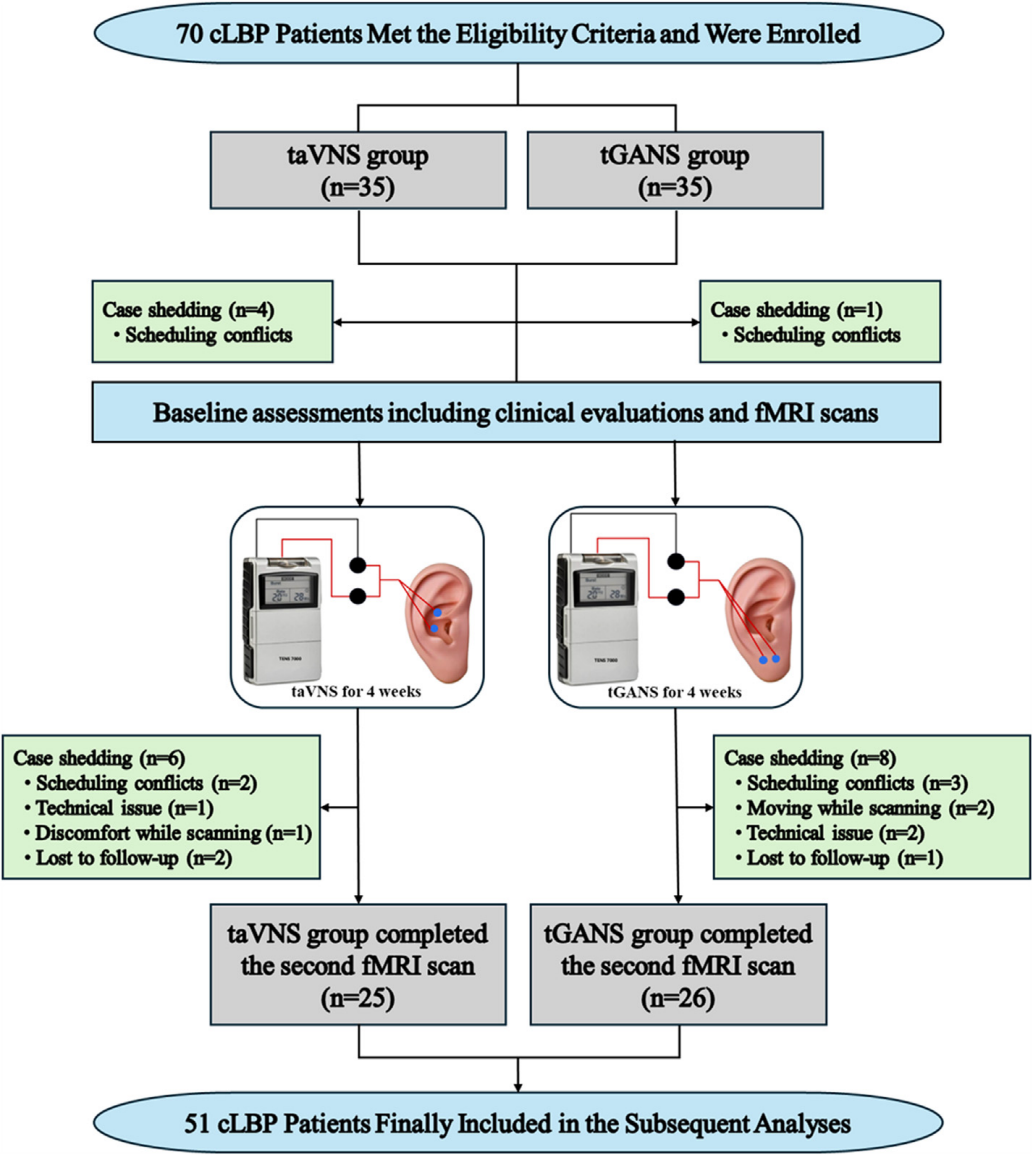
Participant flow diagram. Abbreviations: cLBP, chronic low back pain; taVNS, transcutaneous auricular vagus nerve stimulation; tGANS, transcutaneous greater auricular nerve stimulation; fMRI, functional Magnetic Resonance Imaging.

Intervention: All stimulations were administered using a transcutaneous electrical nerve stimulation device (TENS 7000) with specialized ear electrodes (Bio-Medical Instruments Alpha-Stim CE3). Treatments were self-administered by participants over four weeks (five sessions per week, 30 ​min per session). For the taVNS group, the electrodes were positioned at the auricular concha cymba and concha cavum, while for the tGANS group, electrodes were positioned on the earlobe (Fig. 1). Stimulation frequency was set at 20 ​Hz for all participants, and intensity was adjusted individually to a level described as “moderate to strong but still tolerable.”

After the initial onsite training session (first treatment), participants administered subsequent treatments at home with support from experienced research staff. Research staff conducted follow-up sessions via Zoom after the first treatment until the participants were comfortable with independent self-administration. Weekly video calls were conducted throughout the four weeks of treatment to confirm proper electrode placement, address questions, monitor adherence to the protocol, and assess participant well-being. Participants recorded the date and intensity of each treatment in a diary. Detailed stimulation parameters and positioning are provided in the supplementary material.

The primary clinical outcome was the intensity of low back pain over the past seven days, measured using a visual analog scale (VAS) ranging from 0 to 10. Secondary outcomes included the pain bothersomeness scale score [18,22], the Roland-Morris Disability Questionnaire (RMDQ) [23], the Patient-Reported Outcomes Measurement Information System (PROMIS-29) [24,25], and the Beck Depression Inventory (BDI-II) score [26].

### fMRI data acquisition and preprocessing

All fMRI data were acquired at the Martinos Center for Biomedical Imaging using a 3.0 ​T ​S whole-body scanner with a 32-channel radio-frequency head coil. Each session encompassed two scanning sequences: (1) functional data were acquired using a T2-weighted echo-planar imaging (EPI) sequence with the following parameters: voxel size: 3 ​× ​3 ​× ​3 ​mm^3^, repetition time: 3000 ​ms, echo time: 30 ​ms, slice thickness: 2.6 ​mm, flip angle: 90°, and 44 slices. A total of 164 ​vol were collected. Participants were instructed to wear earplugs, stay awake, keep their head still, keep their eyes open, and blink normally during the resting-state fMRI scan. (2) Structural brain images were obtained using a T1-weighted three-dimensional multi-echo magnetization prepared rapid gradient-echo (MPRAGE) sequence with the following parameters: repetition time: 2500 ​ms, echo time: 1.69 ​ms, slice thickness 1 ​mm, flip angle: 7°, voxel size: 1 ​× ​1 ​× ​1 ​mm^3^ and 176 slices covering the whole brain.

Functional data preprocessing, sFC, and dFC analysis were performed using the SPM12-based toolbox CONN 21a (http://www.nitrc.org/projects/conn). The midbrain/brainstem normalization process was improved by utilizing the spatially unbiased infra-tentorial template (SUIT) [27], which allowed for more precise designation of regions of interest for the subsequent extraction of BOLD signals. Detailed preprocessing procedures with the SUIT toolbox are provided in the supplementary material.

### Seed-based static and dynamic functional connectivity analysis

The functional connectivity analysis employing a seed-based approach was performed using the CONN toolbox v21a. We employed a seed-to-voxel approach using seed [regions of interest (ROIs)] including the bilateral PAG with a 3 ​mm-radius sphere (MNI coordinates x ​= ​±4, y ​= ​−26, z ​= ​−14) [[28], [29], [30], [31], [32]], and the bilateral VTA with a 4 ​mm-radius sphere (MNI coordinates x ​= ​−4, y ​= ​−15, z ​= ​−9; x ​= ​5, y ​= ​−14, z ​= ​−8) [33] as seeds.

The first and second-level sFC and dFC analyses were also performed using the CONN toolbox. Detailed procedures are provided in the supplementary material. For the whole-brain analysis, a threshold of uncorrected *p* ​< ​0.005 ​at the voxel level and false discovery rate (FDR) corrected *p* ​< ​0.05 ​at the cluster level was applied. Considering the significant role of the thalamus, hippocampus, insula, ACC, middle cingulate cortex (MCC), superior frontal cortex, middle frontal cortex, postcentral gyrus (PoCG), paracentral gyrus, hypothalamus, amygdala, caudate, and PAG in pain perception & modulation, and reward processing [32,[34], [35], [36], [37], [38]], we also pre-defined these brain regions as ROIs and derived masks by applying the Harvard-Oxford cortical and subcortical structural atlases, in FSL (https://fsl.fmrib.ox.ac.uk/fsl/fslwiki) and AAL atlases (https://www.gin.cnrs.fr/en/tools/aal). Monte Carlo simulations were performed using the 3dFWHMx and 3dClustSim functions implemented in AFNI for the predefined ROIs to correct for multiple comparisons. In particular, the spatial autocorrelation function (ACF) option in AFNI's 3dFWHMx (https://afni.nimh.nih.gov) was employed to compute the intrinsic smoothness, which was then entered to run Monte Carlo simulations by 3dClustSim to determine the minimum cluster size necessary to maintain a type 1 error rate of 5 ​% [39].

### Statistical analysis

Baseline characteristics between the taVNS and tGANS groups were compared using the independent samples *t*-test and χ2 test. Changes in primary and secondary outcomes before and after the treatment within each group were analyzed using the paired *t*-test. Differences between the two groups were tested using mixed factor analysis of variance (ANOVA) with group allocation (taVNS vs. tGANS) and time (pre-treatment vs. post-treatment) as factors to examine treatment responses between groups, age, and gender were also included in the model as covariates of non-interest. To explore the association between changes in FC and the primary clinical outcome, we extracted the average Fisher Z-scores of the brain regions that exhibited significant sFC changes following the treatment of taVNS or tGANS treatment. After, Pearson correlation analyses were conducted to examine the relationships between changes in sFC Z-scores and changes in the scores of low back pain intensity. The results of the correlation analyses were corrected for multiple comparisons by Bonferroni correction.

## Results

### Demographic characteristics and clinical assessment

A total of 51 subjects (25 in the taVNS group and 26 in the tGANS group) underwent two MRI scans (one before and one after the 4-week treatment period) and were included in the analyses. For details on participant dropout, see the flow diagram (Fig. 1) and the supplementary material.

No significant difference was observed between the two groups regarding age, gender, or baseline scores for the primary outcome and secondary outcomes including RMDQ, pain bothersomeness, and subscores associated with physical, mental, and social health in PROMIS 29 and BDI-II (all *p* ​> ​0.05) (Table 1).

**Table 1:** Demographic and clinical information of each group.

Items	taVNS group (n ​= ​25)	taGNS group (n ​= ​26)	taVNS vs. taGNS	
			T/X^2^	*p*
Age	38.64 (14.54)	41.73 (14.10)	−0.77	0.445
Gender (female/male)	14/11	14/12	0.024	0.877^†^
Pain intensity	5.68 (1.15)	5.39 (1.10)	0.94	0.352
RMDQ	6.72 (4.52)	6.39 (3.89)	0.28	0.777
Pain bothersomeness	5.88 (1.69)	5.89 (1.73)	−0.01	0.992
PROMIS_pain interference_	55.71 (6.80)	58.02 (5.0)	−1.38	0.173
PROMIS_physical function_	47.81 (8.12)	43.89 (5.82)	1.99	0.052
PROMIS_anxiety_	50.14 (8.34)	50.59 (9.92)	−0.17	0.862
PROMIS_depression_	47.91 (8.33)	48.05 (8.99)	−0.06	0.952
PROMIS_fatigue_	53.67 (8.25)	53.68 (11.58)	−0.003	0.997
PROMIS_sleep disturbance_	52.90 (7.19)	53.09 (10.59)	−0.07	0.941
PROMIS_social satisfaction_	48.05 (9.39)	47.45 (8.02)	0.25	0.806
BDI-II	7.76 (6.27)	8.35 (7.13)	−0.31	0.757

Notes: †, the p value was obtained by chi-square test; other p values were obtained by a two-sample *t*-test; RMDQ, Roland-Morris Disability Questionnaire score; BDI-II, Beck Depression Inventory.

Within-group comparisons (before and after taVNS/tGANS treatment) revealed significant reductions in patients' pain-related scores, including pain intensity, RMDQ, pain bothersomeness, and pain interference from PROMIS 29 (For taVNS, *p*_pain intensity_ < 0.001, *p*_RMDQ_ = 0.018, *p*_pain bothersomeness_ ​= ​0.011, *p*_pain interference_ ​= ​0.016; for tGANS *p*_pain intensity_ <0.001, *p*_RMDQ_ = 0.027, *p*_pain bothersomeness_ < 0.001, *p*_pain interference_< 0.001). Additionally, tGANS was associated with significant improvements in physical function and social satisfaction evaluated by PROMIS 29 (*p*_physical function_ ​= ​0.008, *p*_social satisfaction_ ​= ​0.008) (Table 2).

**Table 2:** Clinical outcome measurements in the taVNS and tGANS groups.

Clinical outcomes	tGANS Group (n ​= ​26)				taVNS Group (n ​= ​25)				Between subjects effects group	Interaction effect time∗group
	Pre-treatment mean (sd)	Post-treatment mean (sd)	T	*p*	Pre-treatment mean (sd)	Post-treatment mean (sd)	T	*p*		
Pain intensity	5.39 (1.10)	3.58 (1.33)	6.05	<0.001∗∗	5.68 (1.15)	4.36 (1.66)	4.06	<0.001∗∗	F _(1,47)_ ​= ​2.99, p ​= ​0.09	F _(1,47)_ ​= ​1.31 p ​= ​0.26
RMDQ	6.39 (3.89)	4.50 (4.14)	2.34	0.027∗	6.72 (4.52)	5.28 (4.23)	2.53	0.018∗	F _(1,47)_ ​= ​0.274, p ​= ​0.603	F _(1,47)_ ​= ​0.201, p ​= ​0.656
Pain bothersomeness	5.89 (1.73)	3.58 (1.42)	6.36	<0.001∗∗	5.88 (1.69)	4.64 (1.85)	2.77	0.011∗	F _(1,47)_ ​= ​1.90, p ​= ​0.175	F _(1,47)_ ​= ​0.73, p ​= ​0.397
PROMIS _pain interference_	58.02 (5.0)	53.65 (6.79)	4.69	<0.001∗∗	55.71 (6.80)	52.41 (7.75)	2.60	0.016∗	F _(1,47)_ ​= ​1.01, p ​= ​0.320	F _(1,47)_ ​= ​0.61, p ​= ​0.441
PROMIS _physical function_	43.89 (5.82)	46.77 (7.27)	−2.91	0.008∗	47.81 (8.12)	48.09 (7.85)	−0.33	0.745	F _(1,47)_ ​= ​1.51, p ​= ​0.225	F _(1,47)_ ​= ​5.08, p ​= ​0.029∗
PROMIS _anxiety_	50.59 (9.92)	49.67 (10.50)	0.69	0.499	50.14 (8.34)	49.23 (9.19)	0.53	0.604	F _(1,47)_ ​= ​0.15, p ​= ​0.700	F _(1,47)_ ​= ​0.002, p ​= ​0.964
PROMIS _depression_	48.05 (8.99)	49.06 (9.31)	−1.24	0.228	47.91 (8.33)	47.28 (7.70)	0.43	0.671	F _(1,47)_ ​= ​0.29, p ​= ​0.592	F _(1,47)_ ​= ​1.00, p ​= ​0.323
PROMIS _fatigue_	53.68 (11.58)	52.28 (10.42)	1.11	0.278	53.67 (8.25)	52.95 (9.42)	0.49	0.630	F _(1,47)_ ​= ​0.01, p ​= ​0.928	F _(1,47)_ ​= ​0.23, p ​= ​0.635
PROMIS _sleep disturbance_	53.09 (10.59)	53.51 (7.66)	−0.34	0.741	52.90 (7.19)	51.24 (8.15)	2.00	0.057	F _(1,47)_ ​= ​0.69, p ​= ​0.409	F _(1,47)_ ​= ​1.60, p ​= ​0.212
PROMIS _social satisfaction_	47.45 (8.02)	49.58 (8.10)	−2.88	0.008∗	48.05 (9.39)	50.49 (9.39)	−2.00	0.056	F _(1,47)_ ​= ​0.08, p ​= ​0.778	F _(1,47)_ ​= ​0.018, p ​= ​0.893
BDI-II	8.35 (7.13)	8.50 (8.80)	−0.21	0.840	7.76 (6.27)	7.76 (6.43)	0.00	1.000	F _(1,47)_ ​= ​0.19, p ​= ​0.667	F _(1,47)_ ​= ​0.0001, p ​= ​0.992

Notes: ∗, results were significant at p ​< ​0.05; ∗∗, results were significant at p ​< ​0.001; RMDQ, Roland-Morris Disability Questionnaire score; PBS, pain bothersomeness scale; PI, pain interference; PF, physical function; SD, sleep disturbance; SS, social satisfaction; BDI-II, Beck Depression Inventory.

Between-group comparisons showed no significant differences in all clinical outcomes, except for a trend suggesting better physical function performance in the tGANS group compared to the taVNS group [F _(1,47)_ ​= ​5.08, *p* ​= ​0.029] (not significant after p-value Bonferroni correction).

The average stimulation intensity chosen by participants was 2.7 (in a range from 2 to 8, units according to the device's scale). Most participants were able to adhere to the treatment protocol based on the report. Three participants missed a single treatment session (completing 19 out of 20 sessions), and one participant missed four sessions (completing 16 out of 20), based on the records provided by the participants.

There were two reports of headache, two of dizziness, three of ear pain, and one of arm tingling during the stimulation. These symptoms were potentially related to the stimulation, but resolved quickly when participants adjusted the intensity or repositioned the ear clips as instructed.

### Static and dynamic functional connectivity results

#### Results using PAG as seed region

**Fig. 2:**
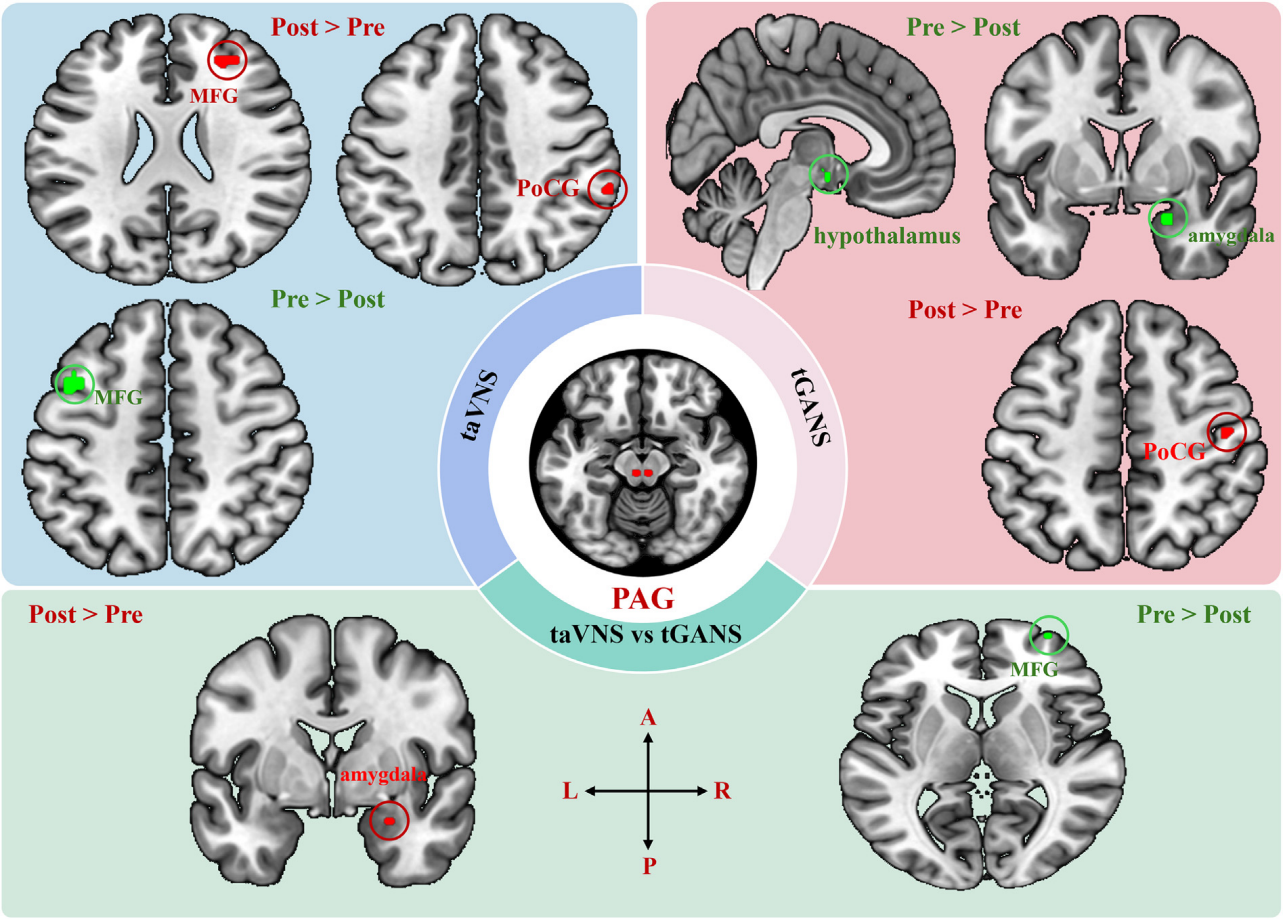
PAG-based resting-state functional connectivity results. Abbreviations: L: left, R: right, A: anterior, P: posterior, PoCG: postcentral gyrus, MFG: Middle frontal gyrus, PAG: periaqueductal gray, taVNS: transcutaneous auricular vagus nerve stimulation, tGANS: transcutaneous greater auricular nerve stimulation. Red represents increased functional connectivity, while green represents decreased functional connectivity after treatment.

Within-group comparisons for taVNS revealed that taVNS was associated with a significant increase in PAG sFC with the left posterior insular cortex, right PreCG, right middle frontal gyrus, left amygdala, MCC, and right supramarginal gyrus/PoCG, as well as a significant decrease in PAG sFC with the left middle frontal gyrus (Table 3, Fig. 2). The dFC analysis showed that taVNS was linked to an increased PAG dFC with the left hippocampus, and a decreased PAG dFC (less variability) with the right anterior insula cortex and right middle frontal gyrus (Table S1).

**Table 3:** Significantly different brain regions identified after taVNS and tGANS treatments in patients with chronic low back pain (cLBP), including age and gender as covariates.

Contrast	ROI	Cluster ID	Peak MNI coordinate			Cluster size	Peak z value	Identified brain regions
			x	y	z			
**taVNS group**								
***post ​> ​pre***	PAG	1	−42	−10	2	46	3.25	L posterior insula cortex∗
		2	48	0	56	125	3.79	R precentral gyrus∗
		3	28	38	24	59	4.3	R middle frontal gyrus∗
		4	−20	−4	−22	15	3.17	L amygdala∗
		5	6	14	32	16	3.75	Middle cingulate cortex ∗
		6	60	−36	44	88	3.23	R supramarginal gyrus/Postcentral gyrus∗
	VTA	1	4	38	18	169	3.78	Bil anterior cingulate cortex∗
		2	−6	−22	−10	69	3.83	L periaqueductal gray∗
		3	−14	18	2	18	3.28	L caudate∗
***pre ​> ​post***	PAG	1	−42	12	48	108	3.88	L middle frontal gyrus∗
	VTA	1	30	−76	12	319	3.8	R superior occipital gyrus
		2	−32	−90	18	404	3.55	L middle occipital gyrus
		3	62	0	38	177	3.32	R precentral gyrus/Premotor cortex
**tGANS group**								
***post ​> ​pre***	PAG	1	46	−16	50	24	3.28	R postcentral gyrus∗
	VTA	1	28	48	42	61	3.71	R superior frontal gyrus∗
		2	4	62	6	60	3.36	R medial prefrontal cortex∗
		3	10	−24	72	30	3.34	R paracentral gyrus/precentral gyrus∗
		4	−12	−30	6	13	3.47	L thalamus∗
***pre ​> ​post***	PAG	1	22	2	−24	41	3.61	R amygdala/parahippocampus gyrus∗
		2	4	−4	−12	46	3.58	Bil_ hypothalamus∗
	VTA	No regions survive the threshold						
**taVNS group vs. tGANS group**								
***post ​> ​pre***	PAG	1	−2	−28	74	18	3.17	L paracentral gyrus∗
		2	18	−12	−24	49	3.49	R Amygdala/Hippocampus∗
	VTA	1	−8	−58	24	372	3.65	Bil_ Precuneus/Posterior cingulate cortex
		2	−12	36	−10	108	3.78	L anterior cingulate cortex∗
***pre ​> ​post***	PAG	1	34	62	2	44	3.64	R medial prefrontal cortex∗
	VTA	1	−62	2	24	304	3.77	L precentral gyrus/Premotor cortex
		2	0	50	38	92	4.5	Bil medial prefrontal cortex∗
		3	−2	30	60	59	4.31	L medial prefrontal cortex∗
		4	−2	−22	60	63	3.41	L paracentral gyrus∗
		5	−2	−34	56	43	3	L paracentral gyrus∗
		6	−6	−34	68	66	3.8	L paracentral gyrus∗
		7	24	−34	60	45	3.74	R postcentral gyrus∗
		8	42	−34	62	59	3.37	R postcentral gyrus∗
		9	66	−28	38	134	3.8	R postcentral gyrus∗

Notes: ∗, results were significant at cluster p ​< ​0.05 after 3dFWHMx and 3dClustSim correction. Other results were significant at cluster p FDR <0.05 corrected at the whole brain level. L, left; R, right; Bil, bilateral; PAG, periaqueductal gray; VTA, ventral tegmental area.

Within-group comparisons for tGANS indicated that tGANS was associated with a significant increase in PAG sFC with the right PoCG, and a significant decrease in PAG sFC with the right amygdala/parahippocampal gyrus and bilateral hypothalamus (Table 3, Fig. 2). The dFC analysis revealed that tGANS led to a decrease PAG dFC (less variability) with the right PreCG (Table S1).

Between-group comparisons showed that taVNS was associated with a greater decrease in PAG sFC with the right medial frontal gyrus than tGANS. Furthermore, tGANS resulted in a greater decrease in PAG sFC with the left paracentral gyrus and right amygdala/hippocampus compared to taVNS (Table 3, Fig. 2). Between-group dFC analysis demonstrated that taVNS was linked to a decreased PAG dFC (less variability) with the bilateral mPFC and right amygdala, while tGANS led to a decreased PAG dFC (less variability) with the left PreCG (Table S1).

#### Results using VTA as seed region

**Fig. 3:**
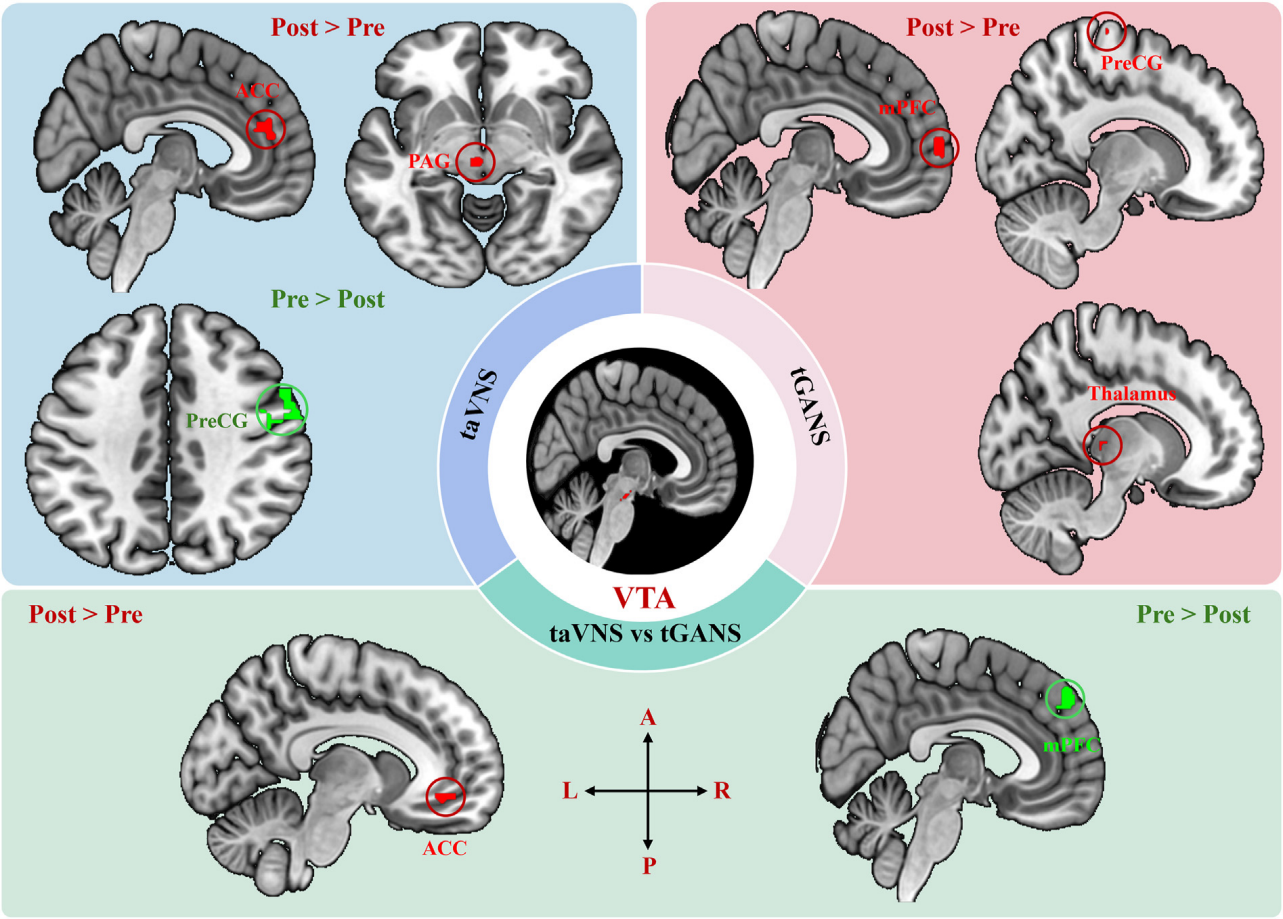
VTA-based resting-state functional connectivity results. Abbreviations: L: left, R: right, A: anterior, P: posterior, ACC: anterior cingulate cortex, PAG: Periaqueductal Gray, PreCG: precentral gyrus; mPFC: medial prefrontal cortex, VTA: ventral tegmental area, taVNS: transcutaneous auricular vagus nerve stimulation, tGANS: transcutaneous greater auricular nerve stimulation. Red represents increased functional connectivity, while green represents decreased functional connectivity after treatment.

Within-group comparisons for taVNS showed a significant increase in VTA sFC with the bilateral ACC, left PAG, and left caudate. Conversely, there was a significant decrease in VTA sFC with the right precentral gyrus (PreCG)/premotor cortex, right superior occipital gyrus, and left middle occipital gyrus (Table 3, Fig. 3). The dFC analysis revealed that taVNS was associated with a significant increase in VTA dFC with the left superior frontal gyrus, and a decrease in dFC (less variability) between the VTA and the left hippocampus (Table S1).

Within-group comparisons for tGANS indicated that tGANS was related to an increase in VTA sFC with the right superior frontal gyrus, right mPFC, right paracentral gyrus/preCG, and left thalamus (Table 3, Fig. 3). There was no significant decrease in VTA sFC detected. The dFC analysis showed an increased VTA dFC with the bilateral middle frontal gyrus, and a decreased VTA dFC (less variability) with the right thalamus and left poCG (Table S1).

Between-group comparisons revealed that taVNS treatment resulted in a greater decrease in VTA sFC with the bilateral mPFC, left PreCG/premotor cortex, left paracentral gyrus, and right PoCG compared to tGANS treatment. Additionally, tGANS treatment was associated with a greater decrease in VTA sFC with the bilateral precuneus/posterior cingulate cortex (PCC) and left ACC compared to taVNS (Table 3, Fig. 3). The between-group dFC analysis demonstrated that tGANS was associated with a greater decrease in VTA dFC with the right thalamus (Table S1).

### Exploratory correlation analysis results

Exploratory analyses were performed to investigate the associations between FC changes and back pain intensity changes. In the taVNS group, a negative correlation was observed between changes in VTA-right superior occipital gyrus sFC and changes in pain intensity (r ​= ​−0.52, p ​= ​0.008) and between VTA-left middle occipital gyrus sFC (r ​= ​−0.47, p ​= ​0.017).

**Fig. 4:**
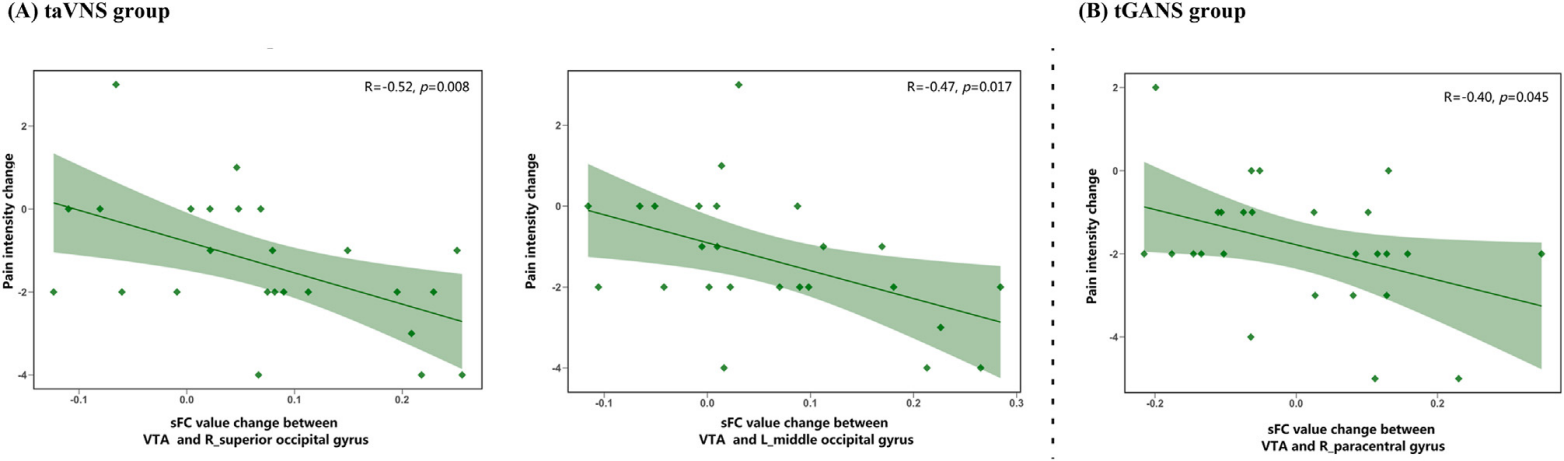
Correlation between changes in static functional connectivity and clinical outcomes in taVNS and tGANS groups. Abbreviations: RMDQ: Roland-Morris Disability Questionnaire; ACC: anterior cingulate cortex; PoCG: postcentral gyrus; PreCG: precentral gyrus; mPFC: medial prefrontal cortex; VTA: ventral tegmental area; PAG: periaqueductal gray; taVNS: transcutaneous auricular vagus nerve stimulation; tGANS: transcutaneous greater auricular nerve stimulation; sFC: static functional connectivity.

In the tGANS group, a significant correlation was found between changes in VTA-right paracentral gyrus/preCG sFC and changes in pain intensity (r ​= ​- 0.40, p ​= ​0.045) (Fig. 4).

## Discussion

We investigated the modulatory effects of taVNS and tGANS on the descending pain modulation system and the reward/motivation network in individuals with cLBP. Results showed that both interventions produced significant pain relief. Functional connectivity analyses showed that both taVNS and tGANS increased PAG sFC with the postcentral gyrus and modulated connectivity between the VTA and regions in the mesocorticolimbic pathways. Between-group comparisons indicated that tGANS was associated with decreased sFC between the PAG and the right amygdala, as well as increased sFC between the VTA and the left precentral gyrus, compared to taVNS. Overall, our findings suggest that both taVNS and tGANS can alleviate chronic low back pain through distinct yet overlapping neural pathways.

### Effects of taVNS and tGANS on cLBP relief

We found that both taVNS and tGANS can provide pain relief for individuals with cLBP. This finding is partly consistent with a prior retrospective single-arm study investigating the effects of taVNS on chronic back pain patients [7], which demonstrated that taVNS significantly reduced back pain intensity.

To further assess the effects of auricular nerve stimulation with other interventions, we calculated the effect sizes of taVNS and tGANS (pre vs post treatment) and compared it with the effect sizes of acupuncture and exercise, which are nonpharmacologic treatments recommended as initial interventions for patients with cLBP in the clinical practice guidelines of the American College of Physicians [40].

In a previous large-scale clinical trial, the investigators evaluated the effects of acupuncture on cLBP, showing that the effect sizes for pain bothersomeness were 0.62, 0.71, 0.79, and 0.28 for individualized acupuncture, standardized acupuncture, simulated acupuncture, and usual care, respectively [41]. In another study, we found that the effects of acupuncture in cLBP treatment demonstrated effect sizes of 1.39 and 0.75 in pain bothersomeness, and 0.96 and 0.74 in pain intensity for the real acupuncture group and the sham acupuncture group [42], respectively. In a study involving exercise therapy, the authors found the effect size for pain intensity was 2.56 with high-intensity training, and 1.29 with moderate-intensity training [43]. These findings align with our results, where the effect sizes for taVNS and tGANS were 0.92 and 1.48 for pain intensity, and 0.70 and 1.46 for pain bothersomeness, respectively. This suggests that the therapeutic effects of taVNS and tGANS for cLBP are comparable to those of acupuncture and moderate exercise, and may be larger than the usual care [41].

An unexpected finding in this study is that tGANS, commonly used as a control intervention in taVNS research, can also significantly relieve low back pain. A literature review suggests that several studies provide evidence in support of this result. For instance, a previous study have shown that taVNS and tGANS (at the ear lobe) produced comparable hypoalgesic effects on experimental heat pain [44]. Additionally, from the perspective of auricular acupuncture, the ear acupoints subcortex and chuiqian (situated anterior to the earlobe but with the GAN distribution) can produce analgesic effects and alleviate negative mood. Studies have shown that stimulating these points can relieve symptoms in patients with trigeminal neuralgia [45] and surgical patients during the perioperative period [46]. Our results support the potential of tGANS as an additional treatment option for cLBP. Further studies are still needed to validate our findings.

### Neural anatomy associated with taVNS and tGANS at earlobe

Literature suggests that the auricular branch of the vagus nerve mainly constitutes somatosensory fibers rather than a visceral sensory fiber. Thus, sensory conduction elicited by taVNS is more likely to terminate in the caudal part of spinal trigeminal nucleus rather than be relayed to the nucleus tractus solitarius (NTS) directly. Furthermore, this branch also contains general somatic afferent fibers from the glossopharyngeal nerve, which similarly terminate in the spinal nucleus of the trigeminal nerve [[47], [48], [49], [50]]. This spinal trigeminal nucleus is located close to the NTS and these two structures are bidirectionally connected [51]. This dual connection could further extend to other brainstem regions (including locus coeruleus and PAG) and higher brain regions, including the lateral and paraventricular nuclei of the hypothalamus, the amygdala, insula, mPFC, and other cortical regions. This could explain why taVNS might modulate the activity in these regions.

The earlobe has been extensively used as a control stimulation site for taVNS as it is free of vagal afferent innervation and is primarily innervated by the greater auricular nerve (GAN). Literature indicates that tGANS may activate several ascending pathways from the spinal cord to the brain, including the spinothalamic, spinolimbic, and spinoparabrachial tracts [52]. The spinoparabrachial tract, in particular, relays sensory information to the parabrachial region, including the locus coeruleus. This activation stimulates catecholaminergic neurons that project to the PAG, potentially modulating nociceptive transmission through the DPMS. Thus, earlobe electric stimulation is not physiologically inert [53]. A previous study found the deactivation in several limbic system regions, including the hippocampus and posterior cingulate gyrus, following either taVNS treatment or tGANS applied at the earlobe [54].

### Common FC alterations induced by taVNS and tGANS

We observed increased sFC between the PAG and PoCG in both the taVNS and tGANS groups, suggesting a shared neurological mechanism underlying these interventions in cLBP patients. The PAG, a key region in the DPMS, plays a critical role in the development and maintenance of chronic pain states, and has been identified as a possible predictor of acute pain chronification [32,55] and non-pharmocological treatment of chronic pain [18,56]. Stimulation of the PAG has even been utilized for managing chronic neuropathic pain [57,58].

Dominant tracts between the PAG and PoCG have also been identified [59], providing a potential structural foundation for the observed functional alterations. The PAG serves as a pain-regulating hub and the PoCG as a sensory receiver. We speculate that the enhanced connectivity between the two regions may represent an increased feedback, which could enhance pain modulation and relieve low back pain.

### Distinct networks induced by taVNS or tGANS

In this study, we utilized both sFC and dFC methods to analyze brain activity. sFC measures the constant, averaged functional relationships between different brain regions, providing a snapshot of how brain areas consistently interact. It is useful for identifying stable and static connections within the brain [60]. Partricularly, dFC captures the fluctuations and variations in these relationships over shorter time windows within the scan. This approach reveals how brain connectivity dynamically changes, offering a more detailed and temporal understanding of brain network interactions [61,62]. By combining sFC and dFC, researchers can provide a comprehensive view of both stable and transient connectivity patterns, enhancing the understanding of how brain networks adapt and reconfigure over time.

### Moduation effects of auricular nerve stimulation on extended PAG network

We found that taVNS is primarily associated with enhanced PAG sFC. For example, we found increased PAG- left amygdala connectivity. The amygdala, a key region of DPMS, has dense reciprocal connections with the PAG. These interactions are essential for the neural processing of nociception, especially in the emotional-affective dimension of pain [63,64]. The increase of the PAG - left amygdala sFC may reflect the increased antinociceptive effects of taVNS.

We also observed increased PAG – MCC. The MCC is known to play a role in the affective aspect of pain. The finding is also consistent with our previous study on taVNS in migraines in which we detected a comparable change in FC between the PAG and MCC [65].

Our results showed that the sFC between the PAG and the right middle frontal gyrus increased, while the sFC with the left middle frontal gyrus decreased following taVNS. Literature suggests that during acute pain stimulation, activation in the middle frontal gyrus occurs predominantly in the right hemisphere, indicating a rightward attentional system designed to respond to pain [66]. The divergent FC changes observed may reflect resource allocation induced by taVNS, which recruits more attentional resources for pain processing, accounting for the opposing trends in the middle frontal gyrus. In addition, we found this strengthened connection between the PAG and the right middle frontal gyrus was less variable as indicated by decreased dFC between them; the finding further endorsed the role of PAG - right middle frontal gyrus connectivity in taVNS.

In the tGANS group, we observed decreased PAG - right amygdala sFC, whereas in the taVNS group, PAG sFC with the left amygdala was increased. In this study, auricular stimulation was applied bilaterally, and bilateral PAG was used as the seed region for functional connectivity analysis. Therefore, it is unlikely that the observed differences in modulatory effects are attributable to the side of stimulation or seed selection. Thus, the findings suggest that tGANS and taVNS may differentially influence the amygdala and the descending pain modulatory system.

The amygdala is bilateral structure located within the limbic system. Traditionally recognized for its role in emotional processing and the assignment of emotional valence to memories and experiences, the amygdala has been extensively studied in the context of fear conditioning and affect. More recently, it has also been implicated in the processing of pain and the modulation of pain. Although still under investigation, studies suggest that the right amygdala appears to exhibit a predominantly pro-nociceptive function, whereas the left amygdala has often been characterized to have no effect on pain modulation, a dampened pro-nociceptive function, or most recently an anti-nociceptive function. Specifically, increased neural activity and excitability in the right amygdala are associated with pain facilitation, whereas enhanced excitability in the left amygdala is linked to pain relief [67]. Based on these hypotheses, we speculate that decreased connectivity between the PAG and the right amygdala may result in blunted pain processing, while increased connectivity between the PAG and the left amygdala may enhance anti-nociceptive function. Ultimately, both mechanisms could contribute to pain relief. Future studies are needed to validate these hypotheses.

In addition, the sFC between the PAG and hypothalamus decreased following tGANS. The two regions are anatomically and functionally interconnected, with significant projections from the hypothalamus to the PAG that facilitate its activation. Stimulation of the hypothalamus can excite PAG neurons [68]. Therefore, the decreased connection between the PAG and hypothalamus may reduce pain perception.

### Moduation effects of auricular nerve stimulation on extended VTA network

We observed increased VTA sFC with the bilateral ACC and left PAG after taVNS. Pain is reported to induce higher inhibitory drive and decreased excitability of VTA dopamine neurons [69], leading to impaired motivated behavior [70]. The ACC, however, has the potential to exert direct top-down control over dopaminergic subcortical regions, including the VTA, via monosynaptic glutamatergic afferent projections, which are pivotal in shaping motivation and reward processes [[71], [72], [73]]. The increased connectivity between the ACC and VTA may promote dopamine transmission and release. In turn, sufficient dopamine may exert an anti-pain effect or an influence on cognitive factors to affect pain [74].

The VTA also interacts closely with the PAG. Dopamine neurons in the VTA target the PAG [75], and synapses from the PAG to the VTA linked to the opioid system have been identified as well [76]. These two systems are neuroanatomically interconnected and interact intricately in pain modulation [77]. Our results indicate that taVNS strengthens this connection, activating both systems to contribute to pain relief.

Decreased sFC was observed between the VTA and the right preCG/premotor gyrus after taVNS. Diffusion tensor imaging study showed a direct anatomical connection between the VTA and motor cortex [78]. Primary motor cortex has been used as a neuromodulation target for pain relieve [79]. In the taVNS group, decreased connectivity between the VTA and motor cortex may be related to movement recovery. As pain intensity diminishes, movement restrictions are alleviated, reducing the demand for motorial motivation.

We also detected a decreased dFC between the VTA and the left hippocampus after taVNS, indicating the variance of VTA-hippocampus FC reduced. The hippocampus is also reported to receive dopaminergic innervation [80]. A previous study found that stimulation on the hippocampus could excite VTA dopamine neurons, and this transsynaptic link might provide an infrastructure for regulating reward-motivated behavior [81]. This alteration may reflect a consistent connection between the two regions.

We only observed increased VTA connectivity after tGANS group. Specifically, we found enhanced VTA sFC with the right mPFC. The mPFC, a region involved in both pain and reward systems, receives dopaminergic projections from the VTA [38]. Chronic pain conditions are often associated with hypoactivity of mPFC pyramidal neurons [82,83]. An animal study uncovered that phasic activation of dopamine inputs from the VTA to the mPFC reduced mechanical hypersensitivity during neuropathic pain states [84]. The increased VTA-mPFC sFC may promote dopamine release and putative glutamatergic output, contributing to pain modulation.

Another interesting finding was that the sFC between the VTA and preCG was increased in the tGANS group, which contrasts with the decreased sFC observed in the taVNS group. As previously mentioned, the connection between these regions plays a role in motor learning and contributes to relieving motor disfunction caused by pain. The increased FC in the tGANS group may represent an active mechanism of motor recovery in tGANS.

We also found increased sFC between the VTA and the left thalamus after tGANS. The thalamus plays a crucial role in DMPS, which can be mediated by dopamine [85]. Dopaminergic neurons in the mesolimbic system originate from cell bodies within the VTA, which serves as a primary source of dopamine for the thalamus [86]. The increased connectivity between the VTA and thalamus may enhance the role of dopamine in regulating descending nociceptive pathways. It is worth noting that the dFC between the VTA and the right thalamus decreased, indicating reduced connectivity variations and fluctuations between the two regions, which further endorsed the modulation effects of tGANS on VTA-thalamus connectivity.

### Limitations of the study

This study has several limitations. Firstly, The sample size in this study is relatively small, limiting the generalizability of our findings. Future studies with larger sample sizes are necessary to validate these findings. Secondly, we employed a stimulating frequency of 20 ​Hz based on our previous research, which demonstrated that taVNS at this frequency could effectively alleviate depressive symptoms and modulate sFC in various brain regions such as the amygdala [87], ventral striatum [11], hypothalamus [88], and DMN [11]. Future research is needed to investigate the role of stimulation frequency in modulating clinical outcomes. Finally, the seed regions (PAG and VTA) in the brainstem are relatively small. To address this challenge, we utilized the SUIT (Spatially Unbiased Infratentorial Template) toolbox to obtain more precise anatomical information to accurately define regions of interest for the subsequent extraction of BOLD signals.

## Conclusions

Auricular nerve stimulation, including both taVNS and tGANS, has shown the potential to relieve symptoms in patients with cLBP. These analgesic effects may be mediated by modulation of the descending pain modulatory system and the reward/motivation circuits via distinct yet overlapping pathways. With respect to the DPMS, taVNS tends to increase functional connectivity between the PAG and limbic regions, including the prefrontal cortex and left amygdala. In contrast, tGANS appears to decrease functional connectivity between the PAG and limbic areas such as the right amygdala and hypothalamus. Additionally, both interventions may influence PAG connectivity with the sensorimotor cortex. Regarding the reward/motivation network, taVNS tends to increase connectivity between the VTA and reward-related or limbic regions such as the ACC and caudate, as well as with the descending pain modulation network (PAG), while decreasing VTA connectivity with the sensorimotor cortex. Conversely, tGANS appears to enhance VTA connectivity with both the reward-related limbic regions, including mPFC, and the sensorimotor cortex. Elucidating the underlying pathways associated with tDCS and tGANS may broaden the potential applications of the NIH SPARC (Stimulating Peripheral Activity to Relieve Conditions) initiative in chronic pain management.

## Author contributions

Experimental Design: JK,

Data collection: SR, JK, SH, VS, LC.

Data analysis: TTL, YFW, VS, YYL,

Manuscript preparation: TTL, YYL, YFW, JK, SH, SR.

## Funding source

This study is sponsored by R34 DA046635 through the NIH HEAL Initiative. The content is solely the responsibility of the authors and does not necessarily represent the official views of the National Institutes of Health or its NIH HEAL Initiative.

## Declaration of competing interest

J.K. has a disclosure to report, including equity ownership in startup companies (MNT, BTT) and involvement in a granted patent related to ear vagus nerve stimulation using headphones/earbuds (US 2018/0339148), as well as several pending patents. All other authors declare no conflict of interest.
