# Supplementary material for: Transcutaneous auricular nerve stimulation modulates the functional connectivity of the descending pain modulation system and reward network in patients with chronic low back pain
Source: Neurotherapeutics. 2025 Jun 2;22(5):e00611. doi: 10.1016/j.neurot.2025.e00611 (PMC12491802; doi:10.1016/j.neurot.2025.e00611)
Supplement: Multimedia component 1 [file mmc1.docx]

**Supplementary Material**

**Transcutaneous auricular nerve stimulation** **modulates the functional connectivity of the descending pain modulation system and reward network in Patients with Chronic Low Back Pain**

Tingting Li^1^, Yuefeng Wu^1^, Yuanyuan Li^1^, Sierra Anne Hodges^1^, Sveta Reddy*, Lucy Chen^1^, Valeria Sacca^1^, Jian Kong^1 *^

^1^Department of Psychiatry, Massachusetts General Hospital, Harvard Medical School, Charlestown, MA 02129, USA

*Corresponding author: Jian Kong. JKONG2@mgh.harvard.edu; Tel: +1 617-726-7893

**The eligibility criteria**

Inclusion criteria were: (1) 18–60 years old, (2) presence of cLBP for a duration of at least six months or longer, (3) pain intensity average of at least a 4 on the 0–10 visual analog scale (VAS) during the screening, and (4) having not participated in acupuncture treatment for at least one year.

Exclusion criteria were: (1) specific causes of back pain (e.g., cancer, fractures, spinal stenosis, infections), (2) complicated back problems (e.g., prior back surgery, medicolegal issues), (3) major systemic diseases or history of head injury or coma, (4) possible contraindications for acupuncture (e.g., coagulation disorders, pregnancy) and conditions that might confound longitudinal effects or interpretation of results (e.g., severe fibromyalgia, rheumatoid arthritis), (5) presence of any contraindications to MRI scanning (for example: cardiac pacemaker, metal implants, claustrophobia, pregnancy, inability to lie still in fMRI scanner), and (6) history of substance abuse or dependence based on self-report.

**The stimulation position and parameters of treatment**

- **taVNS treatment**

The points for taVNS are targeted at the auricular concha cymba and concha cavum for application. Patients assumed either a seated position or lay on their sides. After the disinfection of the stimulation points following standard practice, ear clips were attached to the auricular concha cymba and concha cavum. Stimulation parameters will include: (1) density wave adjusted to 20Hz with 500 microseconds (µs) pulse width, (2) intensity adjusted based on the tolerance of the patient (moderate to strong tolerable stimulation without pain), and (3) treatment will be applied 1 time per day, 5 times per week for four weeks (on-site taVNS treatment plus self-administration at home).

- **tGANS treatment**

The electrodes will be positioned on the earlobe, an area devoid of vagus nerve innervation. The remaining procedures conducted in the tGANS group will be identical to those implemented in the taVNS group.

**MRI data acquisition and preprocessing**

All subjects’ fMRI brain imaging data before and after 4 weeks of treatment were acquired at the Martinos Centre for Biomedical Imaging using a 3.0 Tesla Siemens whole-body scanner with a 32-channel radio-frequency head coil. Each session encompassed two scanning sequences: (1) functional data scanning was acquired using a T2-weighted echo-planar imaging (EPI) sequence with the following parameters: voxel size: 3 × 3 × 3 mm3, repetition time: 3,000 ms, echo time: 30 ms, slice thickness: 2.6 mm, flip angle: 90°, and 44 slices, with a total of 164 volumes were collected. Subjects were told to wear earplugs, stay awake, remain head still, keep their eyes open, and blink normally during the resting-state fMRI scan. (2) Structural brain images were obtained employing a T1-weighted three-dimensional multi-echo magnetization-prepared rapid gradient-echo (MPRAGE) sequence with the following parameters: repetition time: 2,500 ms, echo time: 1.69 ms, slice thickness 1 mm, flip angle: 7º, voxel size: 1 x 1 x 1 mm^3^ and 176 slices covering the whole brain.

The midbrain/brainstem normalization process was improved by employing the spatially unbiased infra-tentorial template (SUIT) [1] in order to more precisely designate regions of interest for the subsequent extraction of BOLD signals. We performed the following preprocessing procedures with the SUIT toolbox: (1) All fMRI and anatomical image pairs were first reoriented to standard LPI orientation by applying FSLs fslreorient2std prior to processing; (2) Tissue segmentation was performed on each subject's T1 whole-brain image using SPM12; (3) The cerebellum and brainstem were isolated in the anatomical image with the cropping algorithms; (4) Normalization was performed using the DARTEL engine (diffeomorphic anatomical registration through exponentiated lie algebra) [2] to find the best correspondence with the SUIT template using a nonlinear deformation based on the segmentation maps produced with the isolation algorithm; (5) All images were resampled to a final voxel size of 2 mm × 2 mm × 2 mm. We did not apply the smoothing to the normalized images because of the tiny size and adjacent anatomical location of brainstem nuclei with reference to a previous study [3].

The preprocessing of the whole brain was performed with the CONN toolbox v21a pipeline (<http://www.nitrc.org/projects/conn>). The following pre-processing steps were implemented for MR imaging processing: (1) the removal of the first five volumes; (2) slice timing correction, head motion correction, and outlier detection using ART (<http://www.nitrc.org/projects/artifact_detect>); (3) coregistration was conducted between each subject’s high-resolution anatomical image and the mean of the functional image; (4) coregistered anatomical images were segmented to gray and white matter and cerebrospinal fluid; (5) normalization to the standard Montreal Neurological Institute (MNI) coordinate space [4] applying the deformation field; (6) smoothing with a 6 mm full-width at half-maximum Gaussian kernel; (7) temporal band-pass filter applied to retain frequencies in the 0.008-0.09 Hz; (8) time series from 12 motion parameters (6 motion parameter estimates plus their derivatives) and from areas with cerebrospinal fluid and white matter were used as regressors to minimize the effect of head motion on the estimation of FC for denoising with reference to the benchmark study.

**Static functional connectivity analysis**

The functional connectivity analysis employing a seed-based approach was performed using the CONN toolbox v21a. In accordance with previous studies, we employed a seed-to-voxel approach using regions of interest (ROIs) including the bilateral PAG with a 3 mm-radius sphere (MNI coordinates x = ±4, y = -26, z = -14) [5,6], and the bilateral VTA with a 4 mm-radius sphere (MNI coordinates x = -4, y = -15, z = -9; x = 5, y = -14, z = -8) [7]. The application of the PAG seed that we used in this study has been utilized by our research group and others for various studies on chronic conditions, such as menstrual pain [8], migraine [9], chronic neck and shoulder pain [10], and low back pain [11]. Consequently, we have reason to believe that the PAG coordinates employed in our study are applicable across different pain conditions and populations. Additionally, the coordinates of the bilateral VTA were obtained from a task-based fMRI study that demonstrated enhanced activation in the VTA in response to reward-predicting cues [12].

For the first-level analysis, a correlation map was produced for each subject by extracting the BOLD time series from each brainstem seed separately and calculating the correlation coefficients between the time series in every seed and all other voxels of the whole brain, respectively. The resulting correlation coefficients were then transformed into Fisher Z-scores for the subsequent analysis of variance (ANOVA) in order to enhance normality. For the second-level rsFC analyses using ANOVA, the group was taken as between-subject factors (taVNS, tGANS), time as within-subject factor (pre-treatment, post-treatment), and gender and age were entered as covariates. For the whole-brain analysis, voxel-wise results were considered significant at uncorrected p < 0.005 at the voxel level and false discovery rate (FDR) corrected p < 0.05 at the cluster level.

A threshold of voxel-wise *p* < 0.005 and *p* < 0.05 false discovery rate (FDR) corrected at the cluster level for the whole-brain analysis was applied. Considering the significant role of the thalamus, hippocampus, insula, ACC, middle cingulate cortex (MCC), superior frontal cortex, middle frontal cortex, postcentral gyrus (PoCG), paracentral lobule, hypothalamus, amygdala, caudate, and periaqueduct grey (PAG) in pain perception & modulation, and reward processing [11,13-20], we also pre-defined these brain regions as region of interests (ROIs) and create masks for these ROIS by applying the Harvard-Oxford cortical and subcortical structural atlases in FSL (https://fsl.fmrib.ox.ac.uk/fsl/ fslwiki) and AAL atlases (https://www.gin.cnrs.fr/en/tools/aal). Notably, the mask for the PAG was created using the CONN toolbox v21a. Based on PAG coordinates (1, -29, -10) reported in pain research [21], a 12 mm spherical ROI was generated to encompass the previously identified PAG region while minimizing the risk of false-positive results. Then, Monte Carlo simulations were performed using the 3dFWHMx and 3dClustSim functions implemented in AFNI (https://afni.nimh.nih.gov) for the predefined ROIs to correct for multiple comparisons. In particular, the spatial autocorrelation function (ACF) option in AFNI’s 3dFWHMx was employed to compute the intrinsic smoothness, which was then entered to run Monte Carlo simulations by 3dClustSim to determine the minimum cluster size necessary to maintain a type 1 error rate of 5% [22].

**Dynamic functional connectivity analysis**

In alignment with the preceding studies [13,23], we segmented the temporal course into 18 windows, with each window sliding by 50 seconds for the first-level dynamic analyses, varying in accordance with the duration of the functional scan. Subsequently, Fisher’s z-transformed Pearson’s correlation coefficient was calculated for each sliding time window, comparing the time series of the seeds with that of all other voxels and generating a collection of beta maps associated with sliding windows for each participant. Dynamic FC (dFC) encompassed the assessment of standard deviation in beta values at individual voxels.

At the group level, dFC was conducted utilizing brainstem seeds, incorporating the following approaches: (1) pairwise t-tests contrasting the pre and post-resting state within the respective taVNS and tGANS group, and (2) a mixed-design ANOVA was employed for the second-level analysis, with the group taken as between-subject factor (taVNS, tGANS), time as within-subject factor (pre- resting state, post- resting state), and gender and age entered as covariates. The same ROIs as in the static functional connectivity analysis were applied. Monte Carlo simulations mentioned before were conducted specifically for the designated ROIs to correct for multiple comparisons. For the rest of the brain, the voxel-wise results of the whole-brain analysis were considered significant at uncorrected p < 0.005 at the voxel level and false discovery rate (FDR) corrected p < 0.05 at the cluster level.

**Detailed dropout reasons**

Five participants had to be excluded due to scheduling conflicts to complete the baseline MRI scan. In the taVNS group, two participants cancelled the post-intervention MRI scan due to scheduling conflicts; one participant was excluded due to the technical issue; one participant felt discomfort while scanning and two participants were lost to follow-up. In the tGANS group, three participants cancelled the post-intervention MRI scan because of scheduling conflicts; two participants were excluded due to artifacts in functional images; two participants were because of technical issues and one participant lost to follow-up.

**Table S1. Dynamic functional connectivity analysis results**

| **Contrast** | **ROI** | **Cluster ID** | | **Peak MNI coordinate** | | | | | **Cluster size** | | **Peak z value** | | **Identified brain regions** | | |
| --- | --- | --- | --- | --- | --- | --- | --- | --- | --- | --- | --- | --- | --- | --- | --- |
|  |  |  |  | ***x*** | | ***y*** | | ***z*** |  |  |  |  |  |  |  |
| **taVNS group** |  |  |  | |  | |  | | |  | |  | |  |  |
| ***post > pre*** | PAG | 1 | | -32 | | -40 | | -4 | 16 | | 3.61 | | L Hippocampus | | |
|  | VTA | 1 | | -24 | | 0 | | 62 | 48 | | 3.27 | | L Superior Frontal Gyrus | | |
| ***pre > post*** | PAG | 1 | | 36 | | 24 | | 0 | 39 | | 3.14 | | R Anterior Insula | | |
|  |  | 2 | | 34 | | 32 | | 38 | 32 | | 2.96 | | R Middle Frontal Gyrus | | |
|  | VTA | 1 | | -32 | | -34 | | -4 | 12 | | 2.82 | | L Hippocampus | | |
| **tGANS group** | | | | | | | | | | | | | | | |
| ***post > pre*** | PAG | No region survived the threshold | | | | | | | | | | | | | |
|  | VTA | 1 | | -26 | | 26 | | 38 | 26 | | 2.85 | | L Middle Frontal Gyrus | | |
|  |  | 2 | | 40 | | 32 | | 26 | 43 | | 3.49 | | R Middle Frontal Gyrus | | |
| ***pre > post*** | PAG | 1 | | 4 | | -22 | | 60 | 36 | | 3.41 | | R Precentral Gyrus | | |
|  | VTA | 1 | | 12 | | -16 | | 2 | 12 | | 3.43 | | R Thalamus | | |
|  |  | 2 | | -44 | | -22 | | 50 | 42 | | 3.55 | | L Postcentral Gyrus | | |
| **taVNS group vs. tGANS group** | | | | | | | | | | | | | | | |
| ***post > pre*** | PAG | 1 | | -56 | | 12 | | 18 | 24 | | 3.63 | | L Precentral Gyrus | | |
|  | VTA | 1 | | 6 | | -10 | | 6 | 15 | | 3.72 | | R Thalamus | | |
| ***pre > post*** | PAG | 1 | | 4 | | 54 | | -16 | 91 | | 3.48 | | R Medial prefrontal Cortex* | | |
|  |  | 2 | | 0 | | 48 | | 20 | 53 | | 3.39 | | Bil Medial prefrontal Cortex | | |
|  |  | 3 | | 34 | | 2 | | -22 | 10 | | 3.03 | | R Amygdala | | |
|  | VTA | No region survived the threshold | | | | | | | | | | | | | |

Notes: *, results were significant at cluster p FDR < 0.05 corrected at the whole brain level. Other results were significant at cluster p < 0.05 after 3dFWHMx and 3dClustSim correction.

**Reference:**

1. Diedrichsen J. A spatially unbiased atlas template of the human cerebellum. Neuroimage. 2006;33(1):127–38.

2. Ashburner J. A fast diffeomorphic image registration algorithm. Neuroimage. 2007;38(1):95–113.

3. Wagner G, de la Cruz F, Köhler S, Bär KJ. Treatment Associated Changes of Functional Connectivity of Midbrain/Brainstem Nuclei in Major Depressive Disorder. Sci Rep. 2017;7(1):8675.

4. Ashburner J, Friston KJ. Unified segmentation. Neuroimage. 2005;26(3):839–51.

5. Kong J, Loggia ML, Zyloney C, Tu P, LaViolette P, Gollub RL. Exploring the brain in pain: activations, deactivations and their relation. Pain. 2010;148(2):257–67.

6. Wei SY, Chao HT, Tu CH, Lin MW, Li WC, Low I, et al. The BDNF Val66Met polymorphism is associated with the functional connectivity dynamics of pain modulatory systems in primary dysmenorrhea. Sci Rep. 2016;6(1):23639.

7. Adcock RA, Thangavel A, Whitfield-Gabrieli S, Knutson B, Gabrieli JDE. Reward-Motivated Learning: Mesolimbic Activation Precedes Memory Formation. Neuron. 2006;50(3):507–17.

8. Wei SY, Chao HT, Tu CH, Li WC, Low I, Chuang CY, et al. Changes in functional connectivity of pain modulatory systems in women with primary dysmenorrhea. Pain. 2016;157(1):92–102.

9. Li Z, Liu M, Lan L, Zeng F, Makris N, Liang Y, et al. Altered periaqueductal gray resting state functional connectivity in migraine and the modulation effect of treatment. Sci Rep. 2016;6(1):20298.

10. Yu CX, Li B, Xu YK, Ji TT, Li L, Zhao CJ, et al. Altered functional connectivity of the periaqueductal gray in chronic neck and shoulder pain. Neuroreport. 2017;28(12):720–5.

11. Yu R, Gollub RL, Spaeth R, Napadow V, Wasan A, Kong J. Disrupted functional connectivity of the periaqueductal gray in chronic low back pain. Neuroimage Clin. 2014;6:100–8.

12. Adcock RA, Thangavel A, Whitfield-Gabrieli S, Knutson B, Gabrieli JD. Reward-motivated learning: mesolimbic activation precedes memory formation. Neuron. 2006;50(3):507–17.

13. Sacca V, Zhang Y, Cao J, Li H, Yan Z, Ye Y, et al. Evaluation of the Modulation Effects Evoked by Different Transcutaneous Auricular Vagus Nerve Stimulation Frequencies Along the Central Vagus Nerve Pathway in Migraine: A Functional Magnetic Resonance Imaging Study. Neuromodulation. 2023;26(3):620–8.

14. Frangos E, Ellrich J, Komisaruk B. R. Non-invasive access to the vagus nerve central projections via electrical stimulation of the external ear: fMRI evidence in humans. Brain Stimul. 2015;8(3):624-36.

15. Hachem LD, Wong SM, Ibrahim GM. The vagus afferent network: emerging role in translational connectomics. Neurosurg Focus. 2018;45(3):E2.

16. Kaniusas E, Kampusch S, Tittgemeyer M, Panetsos F, Gines RF, Papa M, et al. Current Directions in the Auricular Vagus Nerve Stimulation I - A Physiological Perspective. Front Neurosci; 2019;13:854.

17. Shen W, Tu Y, Gollub RL, Ortiz A, Napadow V, Yu S, et al. Visual network alterations in brain functional connectivity in chronic low back pain: A resting state functional connectivity and machine learning study. NeuroImage Cli. 2019;22:101775.

18. Tu Y, Jung M, Gollub RL, Napadow V, Gerber J, Ortiz A, et al. Abnormal medial prefrontal cortex functional connectivity and its association with clinical symptoms in chronic low back pain. Pain. 2019;160(6):1308–18.

19. Yu S, Li W, Shen W, Edwards RR, Gollub RL, Wilson G, et al. Impaired mesocorticolimbic connectivity underlies increased pain sensitivity in chronic low back pain. Neuroimage. 2020;218:116969.

20. Zhang B, Jung M, Tu Y, Gollub R, Lang C, Ortiz A, et al. Identifying brain regions associated with the neuropathology of chronic low back pain: a resting-state amplitude of low-frequency fluctuation study. Br J Anaesth. 2019;123(2):e303–11.

21. Linnman C, Moulton EA, Barmettler G, Becerra L, Borsook D. Neuroimaging of the periaqueductal gray: State of the field. Neuroimage. 2012;60(1):505–22.

22. Bennett CM, Wolford GL, Miller MB. The principled control of false positives in neuroimaging. Soc Cogn Affect Neurosci 4. 2009;417–22.

23. Kaiser RH, Whitfield-Gabrieli S, Dillon DG, Goer F, Beltzer M, Minkel J, et al. Dynamic Resting-State Functional Connectivity in Major Depression. Neuropsychopharmacology. 2016;41(7):1822–30.

24. Thompson JM, Neugebauer V. Cortico-limbic pain mechanisms. Neurosci Lett. 2019;702:15–23.

25. Alshami AM. Pain: Is It All in the Brain or the Heart? Curr Pain Headache Rep. 2019;23(12):88.

26. Li T, Zhang S, Kurata J. Suppressed descending pain modulatory and enhanced sensorimotor networks in patients with chronic low back pain. J Anesth. 2018;32(6):831–43.
